# Suicidality and Suicide Prevention in Brazil: A Systematic Review of Reviews

**DOI:** 10.3390/ijerph22081183

**Published:** 2025-07-29

**Authors:** Luiza Wille Augustin, Pamela Rinozi Teixeira, Kairi Kolves

**Affiliations:** 1 Department of Distance Education, Catholic University Center of Santa Catarina, Jaraguá do Sul 89254-430, Brazil; luiza.wa@hotmail.com; 2 Independent Researcher, Florianópolis 88065-033, Brazil; pamelarinoziteixeira@gmail.com; 3 Australian Institute for Suicide Research and Prevention, School of Applied Psychology, Griffith University, Brisbane 4122, Australia

**Keywords:** suicide prevention, suicidal behavior, systematic review of reviews, Brazil

## Abstract

Suicide is a growing public health concern in Brazil, with significant increases in mortality rates over the last decade and disparities among vulnerable populations. This study aimed to systematically synthesize the recent literature reviews on suicidality and suicide prevention in Brazil, providing an overview of key findings, research gaps, and implications for future studies. This systematic review of reviews follows a pre-registered PROSPERO protocol (CRD42024561892). Searches across five databases (MEDLINE, Scopus, CINAHL, SciELO and LILACS) identified 10 eligible reviews, published between 2019 and 2024, including systematic, integrative, narrative reviews, and meta-analyses. The reviews examined populations such as Indigenous peoples, adolescents, university students, older adults, and healthcare professionals. Findings showed that the risk of suicidal behavior was associated with mental health conditions, social vulnerability, and limited access to mental health services. Particularly high suicide rates were observed among Indigenous populations and adolescents. Across reviews, a lack of interventional studies, limited geographical coverage, and the inadequate training of health professionals were recurrent themes. This review highlights the urgent need for culturally sensitive suicide prevention strategies, greater research investment in underserved populations, and improved healthcare training and coordination. These findings aim to support the development of more effective national suicide prevention policies.

## 1. Introduction

Suicide is recognized as a global public health problem, involving over 700,000 deaths annually. This represents 1% of all global deaths as per the World Health Organization [1]. The most recent World Health Organization [2] report on suicide highlights that the Americas is the only region where suicide rates increased in 2000–2019. According to Brazilian Ministry of Health Epidemiological Bulletin [3], suicide mortality increased by 42% between 2010 and 2021. The total number of suicides recorded in 2021 was 15,507 cases; 78% were men. Suicide has become the third leading cause of death among adolescents aged 15 to 19 and the fourth leading cause among young adults aged 20 to 29 [3]. In terms of vulnerable groups, Indigenous populations have a suicide mortality rate approximately three times higher than the national average [3].

Brazil is the largest country in South America, with a population of 203,080,756 [4]. Its territory covers nearly half of the South American continent (47.3%), spanning 8,515,767.049 square kilometers [4]. Brazil has a complex history of colonization by European nations, primarily Portugal, which involved the enslavement of Indigenous peoples and millions of Africans brought through the transatlantic slave trade [5].

According to the Brazilian Ministry of Health [6], the National Suicide Prevention Strategy was launched in 2006 and later expanded through a 2019 law establishing the National Suicide Prevention Plan [7], aiming to integrate suicide prevention into primary healthcare, expand mental health services, and increase public awareness [7]. However, there remains a lack of intersectoral coordination, with contrary public policies, such as the availability of firearms, complicating prevention efforts [8].

Limited research on suicide in Brazil is also concerning, given its relevance as a public health issue. There is a lack of longitudinal investigations and intervention studies beyond the emergency department [9]. Academic publications on suicide remain limited, influenced by taboos and fragile mental health policies, which complicates training and information dissemination [10]. This gap extends to how healthcare professionals approach suicidal behavior, hindering preventive efforts and emphasizing the need for research focused on their education [11]. Advancing suicide research is crucial for developing more effective prevention and care strategies in the country.

However, on a positive note, a PubMed search using Brazil + suicid* revealed significant growth in Brazilian suicide research over the decades. Since 1965, publications have steadily increased, reaching 1840 articles by March 2025, with an annual average of 37.6 and a peak of 187 peer-reviewed publications in 2022. From 1960 to 2020, academic production grew by approximately 17.2%, reflecting rising interest in the topic and efforts to understand risk factors and improve prevention strategies. Given the increasing volume of research on suicide in Brazil, this study aims to systematically review recent reviews to identify key research trends, highlight gaps in the literature, and provide insights for future studies.

## 2. Materials and Methods

### 2.1. Search Strategy and Inclusion Criteria

The review protocol of this review of reviews (frequently also referred to as an umbrella review [12]) was registered in PROSPERO [CRD42024561892] to ensure transparency. The PRISMA checklist was followed for reporting (Supplementary Table S1). Studies were included if they focused on suicidal behavior and prevention in Brazil, were classified as systematic reviews, integrative reviews, narrative reviews, or meta-analyses, and were published recently between January 2019 and July 2024. Articles in English, Portuguese, and Spanish were eligible for inclusion. Studies were excluded if Brazil was included only as part of a broader analysis without a specific focus on the Brazilian context or if they were primary studies, commentaries, editorials, or opinion pieces.

A comprehensive search was conducted in five electronic databases: MEDLINE, Scopus, CINAHL, SciELO, and LILACS. The search strategy included three main concept groups combined using Boolean operators: (1) suicidal behavior and prevention (“suicide” OR “self-harm” OR “self-poisoning” OR “suicide attempt” OR “suicidal ideation”); AND (2) country of interest (“Brazil” OR “Brasil”); AND (3) study type (“review” OR “meta analysis”). The search terms were applied to titles, abstracts and keywords. Additionally, the reference lists of all included studies were manually screened for the relevant literature.

### 2.2. Data Extraction, Quality Checking and Synthesis

Two authors independently screened titles and abstracts to determine eligibility, followed by full-text reviews. Disagreements were resolved through discussion, with a third author consulted when needed. Extracted data included author, title, journal, year, abstract, review type, the number of included studies, outcomes, exposures or interventions, the target population, and main findings. The risk of bias was assessed using the AMSTAR-2 checklist. Due to study heterogeneity, a narrative synthesis was conducted, emphasizing frequent themes and research gaps.

## 3. Results

**Figure 1:**
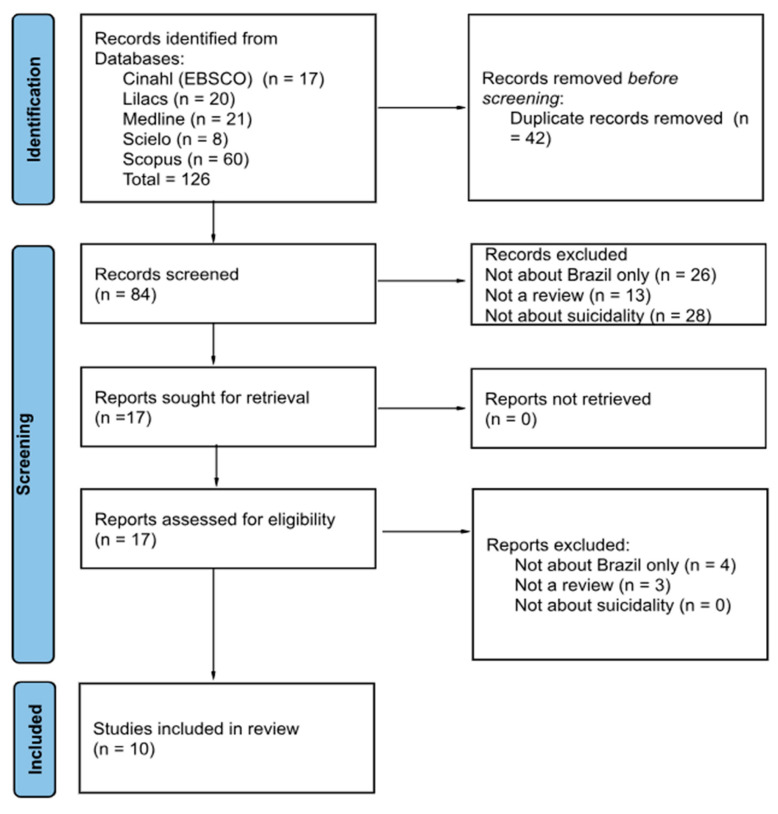
PRISMA flowchart.

The search, conducted in 5 databases, resulted in 126 articles that were screened first for duplicates, then by title, abstract, and full text. Ten papers meet the eligibility for inclusion. Figure 1 shows the PRISMA flowchart. Based on our quality assessment using AMSTAR-2, all reviews had low or critically low scoring (see Supplementary Table S2). Although the technical limitations identified by the AMSTAR-2 tool are important, the consistent low quality seen across reviews may also reflect broader structural challenges in Brazilian suicide research, such as the limited methodological training and funding constraints.

The analysis of the literature on suicide in Brazil revealed a broad range of approaches, integrating both quantitative and qualitative perspectives, and addressing diverse populations and sociocultural contexts (see Table 1 for more details). The 10 included reviews focused exclusively on the Brazilian context and comprised five systematic reviews, three with meta-analyses, one integrative review, and one exploratory review. Five reviews included only quantitative studies, two included only qualitative studies, and three included both. The reviews included a total of 321 distinct papers, after accounting for overlap between reviews (three duplicates). Only two articles focused on the same target group—Indigenous peoples. The results are organized into descriptions of studies.

### 3.1. Description of Reviews

#### 3.1.1. Reviews Including Qualitative Studies

Two reviews including qualitative studies were based on theoretical interpretation and text analysis, exploring philosophical, bioethical, and psychosocial concepts related to suicide [9,10]. An analysis of 20 studies focusing on ethical challenges and the impact of stigma on suicide prevention concluded that suicide remains highly stigmatized, often linked with sin, crime, or mental illness, which negatively affects healthcare responses and patient support [10]. Additionally, they found that professionals often exhibit moralistic and punitive attitudes, highlighting the need for a more humanized and ethical approach in suicide prevention policies. Meanwhile, a theoretical review in psychology was conducted to understand different groups’ conceptions of and discourses on suicide [9]. They identified that psychoanalytic theories dominated the discourse, emphasizing trauma, the symbolic expression of suffering, and the inability to verbalize distress as key elements in suicidal behavior. They also identified that studies tend to focus on post-suicide attempts rather than preventive measures, highlighting a gap in research dedicated to effective intervention strategies and long-term suicide prevention policies.

#### 3.1.2. Reviews Including Quantitative Studies

Five reviews included quantitative studies focusing on trends and factors associated with suicidality [13,14,15,16,17]. A meta-analysis including 44 studies consolidated data on mental disorders among Brazilian university students, concluding that anxiety (37.8%), depression (28.5%), and suicidal behavior (9.1%) were highly prevalent in this population, with female students and those in their early years of study identified as the most vulnerable groups [13]. Meanwhile, an investigation using postmortem studies revealed that 74.7% of suicide victims were men and that 82.9% of cases occurred at home. Based on eight psychological studies, it was identified that psychiatric disorders were present in 90.7% of individuals who died by suicide, and 28.1% had previous suicide attempts [14]. Further, 37 autopsy studies showed that 36.4% had consumed substances before their death, and hanging was the most common suicide method [13].

Similarly, an analysis of 146 epidemiological studies mapped child and adolescent suicidal behavior and ideation in Brazil. The findings revealed a lack of intervention studies, a geographical concentration of research in the South and Southeast regions and significant gaps in data collection on suicidal ideation and attempts among younger populations [15]. A meta-analysis of self-inflicted burns (seven studies) found that self-immolation was linked to higher mortality (RR = 5.1), larger burned areas (MD = 19.2%), and greater risk among women compared to men (RR = 4.0) [16]. Additionally, a systematic review of suicide among Indigenous peoples in Brazil (with seven studies) identified that Indigenous suicide rates (21.8 per 100,000) were nearly four times higher than the national average, particularly among young, single Indigenous men with low education levels [17].

**Table 1:** Description of reviews included.

Authors	Review Type	N of Studies and Years Covered	Target Population	Main Aim	Main Findings
Demenech et al., 2021 [13]	Systematic Review and Meta-Analysis	47 studies (44 included in the meta-analysis) in 2001–2019	Brazilian undergraduate students	Synthesize the prevalence and associated factors of anxiety, depression, and suicidal behaviors among Brazilian undergraduate students.	Suicidal behaviors, including ideation, plans, and attempts, had a prevalence of 9.10% in the undergraduate students population, strongly associated with depression, other mental health disorders, and a family history of suicide.
Gomes et al., 2019 [9]	Integrative Literature Review	17 in 2006–2017	Brazilian populations at risk for suicidal behavior	Analyze the scientific production of Psychology in Brazil related to suicidal behavior, identifying how the subject is addressed and highlighting gaps in prevention and intervention strategies.	The scientific research on suicidal behavior in Psychology in Brazil significantly increased between 2011 and 2013, though it remains limited overall. Most studies used a psychoanalytic approach, often linking suicidal behavior to psychological suffering and existential dilemmas. A critical gap was identified in research on suicide prevention and intervention within the psychosocial care network. Key risk factors for suicidal behavior include social isolation, mental health issues like depression, and a history of suicide attempts. Protective factors, such as family connections, social support systems, and religious involvement, were found to be crucial in reducing suicide risk.
Lima & Nascimento, 2023 [10]	Systematic Review	20 in 2000–2021	General population in Brazil	Map Brazilian academic production on bioethical approaches to suicide, aiming to contribute to debates on ethical conflicts and collaborate in suicide prevention.	The main findings revealed three key concerns: first, stigmatization was found to violate patients human rights and compromise adherence to treatment, thereby undermining suicide prevention efforts. Second, moral dilemmas often involved weighing individual autonomy and dignity against societal, religious, and legal norms. And third, the ethical-political analysis highlighted how suicide reflects structural inequalities and systemic neglect, emphasizing the need for interventions that address these broader sociopolitical and ethical dimensions.
Nascimento et al., 2024 [16]	Systematic Review and Meta-Analysis	7 in 2003–2023	Brazilian individuals with intentional self-inflicted burn injuries	Investigate the profile of intentional self-inflicted burns among Brazilian victims via comparison with non-intentional self-inflicted burns.	Self-inflicted burns made up 9% of the cases analyzed, with women accounting for 64.9% of the victims. Women were significantly more likely to attempt self-immolation than men, with a relative risk of 4.01. Victims of self-inflicted burns had a larger burned surface area (19.2% more) and a higher risk of death (relative risk of 5.13) compared to accidental burn cases. The study emphasized the psychological distress and social vulnerabilities contributing to self-inflicted burns and called for targeted public health strategies to address the issue effectively.
Pereira et al., 2021 [18]	Systematic Literature Review	7 in 2000–2019	Indigenous peoples residing in the Brazilian Amazon	Examine mortality and factors associated with suicide among Indigenous peoples in the Brazilian Amazon.	Suicide rates among Indigenous populations in the Brazilian Amazon are about four times higher than in urban areas. Young males, especially those aged 15 to 21, and single individuals are most at risk. Most suicides occur at home (80.3%), with hanging being the most common method (85.5%). Key risk factors include alcohol use, cultural transitions, and social disintegration. Addressing this issue requires social investigations within villages and the development of culturally appropriate interventions.
Piccin et al., 2020 [15]	Systematic Review	146 in 1966 and 2017 (47.3% published since 2010)	Children (0–9 years) and adolescents (10–19 years) in Brazil, with most studies focusing on adolescents	To systematically review and describe the scientific output on suicide in children and adolescents in Brazil.	Research on child and adolescent suicide in Brazil is limited, with a lack of interventional studies. Most research is observational, using secondary data, and is concentrated in the South and Southeast regions, despite higher suicide rates in the North. There is a critical need for research on suicide prevention and intervention, particularly with culturally tailored approaches for youth. Studies mainly focus on adolescents, with gender-specific findings highlighting vulnerabilities among females, including those related to pregnancy.
Roza et al., 2023 [14]	Systematic Review and Meta-Analysis	45 studies (8 psychological autopsy and 37 autopsy studies) (3 from 1986 to 1988, 42 from 2003 to 2022)	Brazilian individuals who died by suicide, with data derived from postmortem studies	Describe the characteristics of suicide deaths in Brazilian postmortem studies, including psychiatric symptoms, substance use, suicide methods, and demographic data.	Males represented 74.73% of suicide deaths, with hanging being the most common method, followed by poisoning and shooting. Psychiatric conditions were found in 90.67% of cases from psychological autopsies and 35.14% from autopsy studies. Substance use was reported in 36.42% of autopsy cases, and prior suicide attempts were noted in 28.09% of psychological autopsy cases and 23.92% of autopsy cases. Most suicides occurred at the victim’s home (83%). The study highlighted significant gaps in research quality, stressing the need for more robust studies to inform public health policies.
Santos et al., 2019 [19]	Exploratory Literature Review	16 in 2010–2017	Older adults in Brazil, particularly those aged 60 and above, from diverse socioeconomic and cultural backgrounds	Examine publications on suicide among the older adults in Brazil, analyzing characteristics, associated factors, impacts, prevention, and postvention.	The review highlights a high prevalence of suicide among older men compared to older women, which aligns with international trends. Key risk factors identified include depression, chronic illness, social isolation, and the cultural stigmatization of aging. Most suicides among the elderly occurred at home, with hanging and poisoning being the most common methods.
Souza et al., 2020 [17]	Systematic Review	9 in 2006–2019	Indigenous populations in Brazil	Describe the frequency, characteristics, and contributing factors of suicide among Indigenous populations in Brazil.	Suicide rates among Indigenous populations in Brazil are highest among males, single individuals, and those aged 15 to 24 years. Most suicides occur at home and on weekends, with hanging being the predominant method. The main risk factors identified include poverty, poor well-being, social vulnerability, and historical-cultural influences.
Stoppa et al., 2021 [11]	Systematic Review	10 in 2008–2018	Health professionals in Brazil working in public health services across primary care, Psychosocial Care Centers and emergency services	Analyze how public health professionals in Brazil address individuals with suicidal behavior, identifying their practices, conceptions, and challenges	Health professionals in Brazil often lack sufficient training to address suicidal behavior, leading to insecurity and emotional discomfort in managing such cases. Preventive actions include active listening, identifying risk factors, and promoting community awareness. However, barriers such as stigma, inadequate training, limited resources, and fragmented healthcare systems remain. To improve care and prevention, collaboration among multidisciplinary teams and comprehensive training programs are necessary for more effective management of suicidal behavior.

#### 3.1.3. Reviews Including Quantitative and Qualitative Studies

Three studies combined quantitative and qualitative research to gain a more comprehensive understanding of suicide [11,18,19]. One study used quantitative papers to map the prevalence of suicide among Indigenous peoples of the Amazon, combined with qualitative studies of sociocultural factors [18]. Another adopted a similar approach in a review focusing on suicide among older adults, and found that depression, social isolation, chronic illnesses, and loss of autonomy were significant risk factors. Additionally, older men had higher suicide rates, often using more lethal methods, while women had more suicide attempts but with lower lethality [19]. Meanwhile, an investigation examined both quantitative studies on healthcare professionals’ attitudes and qualitative analyses of the challenges faced in assisting individuals with suicidal behavior, finding that many healthcare professionals felt unprepared and insecure when handling suicide cases, often relying on personal judgment rather than established protocols. Stigma, a lack of resources, and insufficient training were also identified as major barriers to effective intervention and follow-up care [11].

### 3.2. Themes

#### 3.2.1. Suicidal Behaviors and Their Associated Factors

The research findings on suicidal behavior in Brazil indicate multiple associated factors and distinct demographic patterns. Three studies [11,13,14] mention psychiatric disorders as relevant factors for suicidal behavior, with depression and schizophrenia being the most frequently reported conditions. Additionally, social stigma was highlighted as an aggravating element, hindering help-seeking and leading to discrimination and inadequate healthcare services [10,11]. Social isolation, family problems, and substance use were also widely cited as risk factors, particularly among Indigenous peoples and the elderly [17,18,19]. Additionally, each specific population studied (categorized by age or ethnic group) had distinct results regarding risk factors, which will be presented next.

#### 3.2.2. Indigenous Populations

Two identified reviews focused specifically on Indigenous suicides and three other reviews mentioned the topic [17,18]. Between the two reviews, a total of 15 studies on suicide among Indigenous populations were reviewed, identifying common patterns. Suicide was more frequent among Indigenous men (73.3%), unmarried individuals (79.5%), and those with low educational attainment—typically between 2 and 11 years of schooling [17,18]. Adolescents aged 15–21 had the highest suicide rates, and most cases occurred at home, with hanging being the predominant method (85.5%) [18]. An overall suicide rate of 40.4 deaths per 100,000 Indigenous individuals was reported, nearly four times higher than the general Brazilian population [18]. The most frequently cited risk factors included family and intergenerational conflicts, alcoholism, social isolation, and socioeconomic issues such as unemployment. Additional contributing factors were also identified, such as the abandonment of Indigenous traditions, abusive consumption of alcohol and other drugs, lack of access to education, youth emotional instability, territorial confinement and resettlements, sexual violence, proximity to urban populations, and cultural fragilization [17].

Although not exclusively focused on Indigenous populations, three other reviews also noted high suicide rates among them. One review highlighted the scarcity of research on this topic [10], while another discussed cultural imposition and territorial overcrowding as risk factors for suicide among young Guarani/Kaiowa individuals [9]. Additionally, some studies addressing Indigenous youth were identified, but no specific findings were presented [15].

#### 3.2.3. Age Groups

Two reviews specifically focused on suicide within defined age groups: one concentrated on children and adolescents, while another examined older adults [15,19]. The review focusing on undergraduate students can also be considered an age group study, as it highlights that most individuals in this population are young adults [13]. These studies explored social and cultural factors influencing suicide risk in their respective age groups. Other reviews included age-related data but did not focus exclusively on one age group [10,11,14].

Suicide is the second leading cause of death among young people aged 15–29, attributed to socioeconomic instability, increased mental health disorders, and social pressure [10]. Similarly, it is the third leading cause of death among individuals aged 10–24, with rising rates linked to bullying, academic pressure, social isolation, and a lack of accessible mental health services [15]. It is also identified as the fourth leading cause of death among young and adult individuals aged 15–30, reflecting alarming patterns in this population segment, especially among men, who are more likely to use lethal methods [14]. Specifically, among Brazilian Indigenous peoples, the highest suicide rates are reported in the age group from 15 to 24 years old [17]. This high mortality is linked to cultural disintegration, alcohol abuse, family conflicts, a lack of access to education, and limited employment opportunities, which are more common in this ethnic group in comparison to the general youth population [17]. Additionally, suicide attempts are one of the leading causes of hospitalization in the Brazilian Unified Health System (SUS) among female adolescents, suggesting a high level of emotional distress related to interpersonal conflicts, trauma, and gender-based violence [9].

The prevalence of suicidal behavior (including ideation, plans, and attempts) among university students was 9.1%, being more frequent among women, individuals with a family history of suicide, and homosexual or bisexual individuals [13]. Among children and adolescents, suicidal ideation ranged from 10.8% to 27.1%, with suicide attempts recorded in up to 9.9% of cases. Suicide rates were the highest among young people in the northern region of Brazil, with hanging being the most common method [15].

Meanwhile, at the other end of the age spectrum, suicide among older adults has been progressively increasing in Brazil, with those aged 60 and older being the most affected [19]. This increase is associated with social isolation, depression, chronic illnesses, financial dependency, and the loss of autonomy [19]. Elderly individuals, especially men, have fewer social support networks and higher adherence to traditional gender roles that discourage them from seeking help, and often use more lethal methods, contributing to the higher suicide mortality rate in this group. Among elderly individuals, the main risk factors were depression, chronic illnesses, financial losses, and difficulty adjusting to retirement, with hanging being the most prevalent method [19].

#### 3.2.4. Professional Approaches and Issues

A fourth theme identified in the reviews refers to professional approaches and ethical issues related to suicide prevention and care. One review [9] focusing on scientific productions in psychology concludes there is a predominance of theoretical–conceptual discussions and a lack of practical strategies for intervention, especially in everyday professional settings. They also highlight gaps in the training of psychologists in terms of dealing with suicidal behavior, indicating the need for greater articulation between academic knowledge and clinical realities [9]. Another review [10] examined Brazilian bioethical publications on suicide, underscoring the moral and ethical tensions involved in the autonomy of suicidal individuals, especially in clinical and institutional contexts. Rather than reducing care to compulsory death prevention practices rooted in surveillance and control, the authors argue that suicidal behavior demands ethical and political engagement with the complex meanings these acts provoke and express. [10]. Finally, a third review [11] focuses specifically on the role of health professionals in Brazil, revealing difficulties in communication, emotional preparedness, and institutional support when dealing with healthcare patients with suicidal behavior. The authors emphasize the importance of offering ongoing training, spaces for professional reflection, and intersectional coordination to enhance the quality of care [11]. Together, these studies reinforce the need to move beyond individual risk factors and consider how the social context, professional practices, ethical dilemmas, and structural limitations shape suicide prevention in Brazil.

## 4. Discussion

The aim of this review was to synthesize recent reviews on suicidality in Brazil, identify research trends, and highlight key gaps in the literature. Although Brazil is the largest country in South America and has expanded its suicide prevention efforts in recent years [7], we identified ten eligible reviews published between 2019 and 2024. These reviews were diverse, particularly in terms of their target populations and research aims.

The heterogeneity of the target populations included Brazilian undergraduate students [13], individuals who sustained self-inflicted burns [15], Indigenous peoples [16], children and adolescents [15], older adults [19], or broader categories such as populations at risk [9]. The studies also varied notably in terms of their main aims. Some focused on estimating the prevalence of suicidal behavior and associated factors among university students [13], older adults [19], and victims of self-inflicted burns [16]. Others aimed to map the national scientific output on suicide to specific populations, such as children and adolescents [15], Indigenous peoples [17,18], or broader thematic contexts, such as the bioethics [10] and psychological research literature [9]. One study reviewed autopsies to analyze demographic, clinical, and behavioral patterns related to suicide deaths [14]. Lastly, an examination was conducted on how health professionals in Brazil deal with suicidal behavior in practice, highlighting the challenges faced across different levels of care [11]. Overall, the studies addressed a wide range of populations and perspectives, but none explored long-term prevention strategies or evaluated the effectiveness of interventions.

Geographical representation was also uneven across the studies, reflecting a broader issue in Brazilian suicide research. According to the Brazilian Ministry of Foreign Affairs [5], Brazil is the fifth largest country in the world and is divided into five major regions: North, Northeast, Center-West, Southeast, and South. Although only a few reviews addressed regional disparities in suicide research, available data suggest a clear concentration of studies in the Southeast, followed by the South. For example, it was reported that 57.4% of studies on suicidal behavior among university students were conducted in the Southeast region [13]. Similarly, 49.3% of studies on child and adolescent suicide originated from the Southeast, with the state of São Paulo alone accounting for 31.5% and Rio Grande do Sul, in the South, accounting for 13.7% [14]. Postmortem studies also followed this pattern, with most cases reported from São Paulo (N = 7527), followed by Rio Grande do Sul (N = 4421) [14].

While most of the research is concentrated in the Southeast, studies focusing on Indigenous populations represent a notable exception. These reviews predominantly addressed communities located in the North of Brazil, particularly in the Amazon region [11,18]. The findings of these studies reveal alarmingly high suicide rates among Indigenous peoples, up to four times higher than the national average, and highlight complex contributing factors such as historical and ongoing processes of cultural disintegration, limited access to education and healthcare and socioeconomic marginalization. Both reviews emphasized the urgent need for culturally grounded and community-led suicide prevention strategies that are developed in partnership with indigenous populations [17,18]. A recent study analyzing suicide among Indigenous populations in Brazil between 2000 and 2020 supports these findings by identifying common contributing factors such as unemployment, limited access to mental health services, low income and land-related conflicts [20]. However, the study also highlights that these factors vary across regions and ethnic groups, reinforcing the need for context-specific approaches.

Beyond the Brazilian context, international literature has emphasized the need to understand suicide through postcolonial, sociocultural, and historical lenses. Studies with Indigenous populations in Canada and Alaska, for example, identify cultural continuity, land rights, and historical trauma as key factors in either exacerbating or mitigating suicide rates [21,22]. In Australia, recent evidence shows that Aboriginal and Torres Strait Islander communities with stronger cultural connectedness (measured by participation in ceremonies, community events, and Indigenous language use) presented significantly lower youth suicide rates, despite facing social and economic adversity [23]. This reinforces the need for culturally tailored approaches to studying and intervening with traditional populations affected by colonization, such as Brazilian Indigenous.

Suicidal behavior in Brazil, as reported across the included reviews, was associated with a range of psychological, social, and structural factors. Mental health disorders were identified as risk factors across different groups, including university students [13], older adults [19], and the general population in postmortem analyses [14]. Substance abuse, particularly alcohol use, also emerged as a relevant factor in studies involving Indigenous populations and the general population [14,17]. Social vulnerability, including unemployment and social isolation, was frequently reported as contributing to increased suicide risk, particularly among marginalized groups such as Indigenous populations [17,18], older adults [19] and, within the general population, men especially [9]. Structural issues, such as stigma surrounding mental illness and suicide, as well as the lack of preparedness among healthcare professionals to address suicidal behavior, were also highlighted [9,11]. These findings underscore the multifactorial nature of suicide and the need for integrated prevention efforts that address both clinical and social determinants.

Among these factors, age-specific vulnerabilities stand out as particularly relevant. Research has emphasized the high burden of suicide among specific age groups in Brazil. Suicide ranks among the leading causes of death for young people aged 10 to 29, with contributing factors including socioeconomic instability, mental health disorders, bullying, academic pressure, social isolation, and limited access to mental health services [10,14,15]. Among Indigenous youth aged 15 to 24, suicide rates are particularly high, driven by cultural disintegration, alcohol abuse, family conflict, educational exclusion, and a lack of employment opportunities [17]. Suicide attempts are also a major cause of hospitalization among female adolescents, often linked to interpersonal violence, trauma, and emotional distress [9]. In university settings, suicidal behavior affects an estimated 9.1% of students, especially among those with a family history of suicide or belonging to vulnerable social groups [13]. These findings point to a broad spectrum of risk factors across youth populations.

In Brazil, suicide rates increase progressively with age. Individuals aged 70 to 79 consistently present the highest rates. Between the ages of 20 and 34, rates have shown an upward trend in recent decades, while the 50 to 69 age group displays variable patterns depending on the region and historical period. Regional data further reveal that younger age groups tend to be more affected in Northern areas, whereas older age brackets carry the highest burden in the South, Southeast, and Central-West regions [24].

Findings from the Epidemiological Bulletin [3] support and expand on this perspective. The bulletin reports that suicide remains one of the leading causes of death among adolescents and young adults, with suicide mortality accounting for 6.9% of deaths among individuals aged 15 to 19 and 5.6% of deaths among those aged 20 to 29. Even in the 5 to 14 age group—where overall mortality is low—suicide accounts for 3.4% of all deaths, indicating a substantial impact. Among older adults, although suicide mortality in not a leading cause of death, absolute suicide rates peak among those aged 70 and older, reaching 18.1 deaths per 100,000 population in this age group [3]. This trend is particularly pronounced among men, and highlights the persistent risk factors in late life, such as social isolation, depression, physical illness, and loss of autonomy [19]. In summary, these data reveal a bimodal pattern: youth face high relative risk, while older adults experience high absolute mortality. This dual burden reinforces the necessity of life stage-specific and culturally appropriate suicide prevention strategies. The alignment between individual-level studies and national surveillance data underscores a need for policies and interventions that are sensitive to the distinct vulnerabilities associated with each phase of the lifespan [3,24].

Despite the clarity around risk groups and patterns, a recurring issue across the reviews was the lack of intervention-focused research. Most studies were observational and descriptive, with limited evaluation of preventive strategies or treatment outcomes. For example, a review of 146 studies on child and adolescent suicide found no intervention-based studies [15]. Similarly, seven other reviews [10,13,14,16,17,18,19] included in this study made no mention of interventional research. Only two reviews [9,11] identified studies with an interventional focus. It was noted that, within the Brazilian psychosocial care network, there is a scarcity of longitudinal research and proposals for ongoing care of individuals with suicidal behavior, beyond emergency hospital-based treatment [9]. Some studies addressing interventions at the primary, secondary, and tertiary levels were also identified [11]. However, most of these studies only provided narrative descriptions of practices and challenges faced by professionals, revealing common issues such as stigma and lack of suicide-specific training. Overall, this reflects a broader limitation in suicide research in the country, where few studies test or implement culturally and contextually appropriate approaches.

To address these issues, structured and ongoing training programs for frontline workers are essential, particularly in primary care. International models, such as the Zero Suicide framework, have shown promising results by integrating systematic screening, safety planning, and continuity of care within health systems [25]. Adapting such evidence-based approaches to the Brazilian context could strengthen national suicide prevention strategy and help reduce stigma and fragmentation in care. However, the implementation of public policies for suicide prevention on Brazilian context faces systemic obstacles, including insufficient funding, a lack of intersectional coordination, and the predominance of reactive rather than preventive measures [26]. These structural, political, and professional challenges help explain the persistent gaps identified in the scientific literature, particularly the scarcity of robust intervention studies and the limited evaluation of suicide prevention strategies in Brazil.

## 5. Conclusions

The findings of this review of reviews highlight high suicide rates among youth and older adults, with Indigenous populations showing particularly severe outcomes. Research remains concentrated in the Southeast, despite regional disparities in suicide burden. Across studies, there is a lack of interventional research, with most focusing on descriptive data. These gaps limit the development of effective, context-specific strategies. There is an urgent need for nationally coordinated suicide prevention efforts informed by regional realities, population-specific vulnerabilities, and evidence-based interventions tailored to the Brazilian context.

## Data Availability

There is no additional data.

## Supplementary Materials

The following supporting information can be downloaded at: https://www.mdpi.com/article/10.3390/ijerph22081183/s1, Table S1: PRISMA Checklist [27]; Table S2: AMSTAR-2 Quality scorings.
